# Application of whole exome sequencing in carrier screening for high-risk families without probands

**DOI:** 10.3389/fgene.2024.1415811

**Published:** 2024-06-24

**Authors:** Qinlin Huang, Zhongjie Wang, Yanling Teng, Wen Zhang, Juan Wen, Huimin Zhu, Desheng Liang, Lingqian Wu, Zhuo Li

**Affiliations:** ^1^ Center for Medical Genetics, Hunan Key Laboratory of Medical Genetics, MOE Key Lab of Rare Pediatric Diseases, School of Life Sciences, Central South University, Changsha, China; ^2^ Laboratory of Molecular Genetics, Hunan Jiahui Genetics Hospital, Changsha, China

**Keywords:** exome sequencing, adverse pregnancy history, consanguinity, high-risk family, carrier screening

## Abstract

**Purpose:**

This study aimed to screen the genetic etiology for the high-risk families including those with an adverse pregnancy history, a history of consanguineous marriages, or a history of genetic diseases, but lack of proband via whole exome sequencing (WES).

**Methods:**

128 individuals from high-risk family were tested by WES. The candidate variants were analyzed according to the ACMG criteria to screen the potential carriers. At-risk couples (ARCs) who harbored the same causative gene were provided with precise fertility guidance to avoid the birth of children with birth defects.

**Results:**

The total detection rate was 36.72%, with pathogenic/likely pathogenic (P/LP) variants found in 47 individuals, and variants of uncertain significance (VUS) were found in 34. Among couples with adverse pregnancy history: P/LP variants were found in 38 individuals, and VUS were found in 26, for a detection rate of 34.55%; among members of family history of genetic disease or consanguineous marriages: P/LP variants were found in nine individuals, and VUS were found in 8, for a detection rate of 50.00%. Otherwise, we detected 19 ARCs who both carried P/LP variants in the same gene, with a theoretical offspring prevalence of up to 7.42%.

**Conclusion:**

In the absence of probands, carrier screening using WES can provide an efficient tool for screening the molecular etiology of high-risk families.

## 1 Introduction

In genetic counseling, families with an adverse pregnancy history, a history of consanguineous marriages, or a history of genetic diseases are usually defined as high-risk families for their higher risk of having a child with birth defects. Adverse pregnancy history concludes a history of miscarriages, stillbirths, congenital disabilities, neonatal or infant deaths, or congenital development-related disorders (Quenby et al., 2021). Families with a history of adverse pregnancy outcomes have a significantly increased risk of recurrent miscarriage or the birth of an affected infant (Glynn et al., 2009). Consanguineous marriages have been found to increase the incidence of intellectual disability, epilepsy, low fertility, miscarriage, and infant and child mortality (Tuncbilek and Koc, 1994; Pedersen, 2000; Bittles and Black, 2010). In addition, offspring with a family history of genetic diseases carry a greater risk of having a child with Mendelian inherited disorders (Hinton, 2008). In such families, the likelihood of a miscarriage or giving birth to a child with congenital disabilities is much higher than that in an average family.

In high-risk families, although chromosomal abnormalities are the most common cause of birth defects (Ohno et al., 1991; Skinner et al., 2003; Rajcan-Separovic et al., 2010; Smits et al., 2020), the genetic etiology of 40%–50% of miscarriage cases with normal karyotypes remains unknown (Sierra and Stephenson, 2006). In 2013, Larsen et al., for the first time, linked miscarriages to pathogenic variants in single or multiple genes (Larsen et al., 2013). Since then, testing for genetic disorders has become a crucial part of detecting adverse pregnancy causes, such as miscarriages.

Recently, next-generation sequencing (NGS), especially whole exome sequencing (WES), has been used to diagnose diseases and verify the causes of miscarriages. In 2021, Najaf et al. performed WES on miscarriage products without chromosomal abnormalities to determine the cause of recurrent miscarriages in consanguineous couples (Najafi et al., 2021). Meanwhile, prenatal WES performed in the presence of a normal fetal karyotype and chromosomal microarray analysis has been demonstrated to detect 20%–80% of pathogenic variants (Drury et al., 2015; Yadava and Ashkinadze, 2017). For high-risk families, the parents of the probands are likely carriers of recessive pathogenic variants (Yang et al., 2017; Petrovski et al., 2019) and are at a higher risk of giving birth to a child with the disease. Notably, several families, for reasons such as the loss of miscarriage products or fetal samples, cannot provide the proband sample information for a precise diagnosis of the causes of adverse pregnancies. At the same time, most of these families still pursue childbirth, and providing precise fertility guidance for them is difficult. Therefore, WES-based carrier screening (CS) may be an excellent aid for the parents of these high-risk families.

CS was first proposed in 1980 (Riordan et al., 1989; Ioannou et al., 2014), and with the extensive application of NGS technology, the CS has been developed into the current expanded carrier screening (ECS), which can screen approximately 200 genes simultaneously. ECS allows the estimation of carrier rates in endemic populations and can serve as an excellent tool to screen for carriers of single-gene disorders. Tong et al. performed exome sequencing of 2234 couples in 2022 (Tong et al., 2022). Overall, 94.5% of them were carriers of at least one disease-causing variant, and at-risk couples (ARCs) who carry pathogenic variants in the same recessive gene ultimately more likely to choose elective options, such as preimplantation genetic testing (PGT), gamete donation, and adoption.

Using WES for CS is an excellent way to detect pathogenic/likely pathogenic (P/LP) variants for couples at a higher risk of having genetically affected children. In 2021, Sallevelt et al. performed WES on 100 consanguineous couples, resulting in the detection of previously unknown P/LP variants in 28 pairs of couples (28%) or their families (Sallevelt et al., 2021). However, few reports exist on CS in high-risk families, and the efficacy of WES in these families is unknown. Therefore, our study aimed to investigate the effectiveness of WES-based CS in high-risk families, especially for those without proband, and to determine the carrier status regarding the causative genes of monogenic disorders in these couples.

## 2 Materials and methods

### 2.1 Subjects

A total of 128 patients from high-risk families at Hunan Jiahui Genetics Hospital were enrolled in this study, including 55 couples with an adverse pregnancy history (i.e., gave birth to, miscarried, or underwent induced labor of an infant with a genetic disease) and without fetal samples (Supplementary Table S1). The remaining 18 patients had a family history of genetic diseases or consanguineous marriages (10 individuals with a family history of genetic disease and 8 with a history of consanguineous marriages; the 8 with a history of consanguineous marriages were all couples, whereas only two individuals with a family history of genetic disorders were a pair of couples) (Supplementary Table S2). All couples sought genetic counseling and genetic testing at Hunan Jiahui Genetics Hospital between 2016 and 2022 due to adverse pregnancies or high-risk fertility. Informed consent was obtained from all subjects. This study was approved by the Ethics Committee of the Center for Medical Genetics, Central South University, Hunan, China (2021-1-26).

### 2.2 Whole exome sequencing

Genomic DNA was extracted from peripheral blood samples using the QuickGene DNA Whole Blood Kit L (FUJIFILM, Tokyo, Japan) according to standard extraction methods. Peripheral blood (1 mL) from each participant was subjected to WES (Berry Genomics Inc., Beijing, China). The peripheral blood samples were stored at 4 °C until further processing if needed. Exons were captured using Nano WES 2.1 (Berry Genomics Inc., Beijing, China) and sequenced using Illumina Novaseq6000 platform (Illumina, San Diego, United States) with 150 bp paired-end reads. Sequencing reads were aligned to the human reference genome (hg18/GRCh38).

### 2.3 Data analysis

Variant interpretation was performed using ANNOVAR software. Among all variants, those rarely seen in populations with minor allele frequencies (MAF) < 1% in exonic, splicing, UTR3, and UTR5 regions were singled out by referring to the population databases, including the 1000 Genomes Project, the Exome Aggregation Consortium (ExAC) project, and the Genome Aggregation Database (gnomAD). Several variants that did not fit the mode of inheritance were excluded. According to the probands’ clinical phenotype or family history, candidate variants that might be associated with the probands’ phenotype or lead to adverse pregnancies were selected for more profound annotation. Pathogenicity prediction of the candidate variants was performed using the computational program Varcards (https://varcards.biols.ac.cn/). The guidelines and standards of the American College of Medical Genetics and Genomics (ACMG) and the Association for Molecular Pathology (AMP) (Richards et al., 2015) were used as references to describe candidate variants. Notably, for couples with an adverse pregnancy history or family history of genetic diseases in our study, only variants related to the probands’ phenotype or family history were reported (which would still be reported if P/LP variants of the same genes were identified in the remaining systems found in the couple). All candidate variants were reported in couples with a history of consanguineous marriage.

### 2.4 Sanger sequencing

Sanger sequencing was performed using DNA extracted from peripheral blood samples to confirm candidate variants. After selecting the candidate variants, we designed primers using Primer Premier five and performed polymerase chain reaction to amplify the variants. Sequencing reactions were performed by Tsingke (Tsingke Biotechnology Co., Ltd., Beijing, China), and the data were analyzed using Lasergene-SeqMan software.

## 3 Results

### 3.1 Initial genetic variant analysis in couples with an adverse pregnancy history

We initially analyzed the WES results of 55 couples with adverse pregnancy histories without fetal samples. The history of adverse pregnancies is shown in Supplementary Table S1. A total of 72 variants were detected, all of which were heterozygous. According to the ACMG guidelines, 42 variants were classified as P/LP variants. In general, P/LP variants were found in 38 individuals, variants of uncertain significance (VUS) were found in 26, with a detection rate of 34.55%. Of the 72 variants, 53 were found in the ClinVar database, and 44 variants were mentioned in previous literature (Table 1). Of the 42 P/LP variants detected, 35 were found in the ClinVar database, and 34 were mentioned in previous literature. We classified all 72 candidate variants according to the pathogenicity-related system, resulting in the highest proportion of metabolism-related variants at 31.9% (n = 23, N = 72), followed by neurological variants at 16.7% (n = 12, N = 72), and the remaining system-related variants had a more even distribution overall (Table 2). Among the 72 variants detected, metabolism-related variants accounted for the highest proportion at 31.9% (n = 23, N = 72), followed by neurological variants at 16.7% (n = 12, N = 72), and the remaining other system-related variants had an overall more even distribution (Table 2). Of the 42 variants classified as P/LP by the ACMG pathogenicity rating system, nearly half (45.2%, n = 19) were metabolic system-associated variants, followed by neurological system-associated variants, which also accounted for a relatively high proportion of variants (14.3%, n = 6), and the number of detected P/LP variants did not vary greatly between the other systems.

**TABLE 1:** Details of the variants identified in couples with an adverse pregnancy history in the present study.

Case ID	Gene and Transcript	Variant	Type of variation	ACMG	Justification^a^	Abnormalities of the proband^b^
Metabolic System						
17,184	*MMACHC*(NM_015506)	c.609G>A:p.W203X	Nonsense	P(PVS1+PM2+PP3+PP4)	PMID: 19370762; 20631720	Methylmalonic acidemia or propionic academia
		c.609G>A:p.W203X	Nonsense	P(PVS1+PM2+PP3+PP4)	PMID: 19370762; 20631720	Methylmalonic acidemia or propionic academia
17,587	*TH*(NM_199292)	c.457C>T:p.R153X	Nonsense	P(PVS1+PM3+PM2_Supporting)	PMID: 28087438; 20056467; 20056467	Phenylketonurics
	*PAH*(NM_000277)	c.782G>A:p.R261Q	Missense	P(PS3+PM3_VeryStrong + PP3+PP4)	PMID: 17935162	Phenylketonurics
18,876	*GUSB*(NM_000181)	c.1918_1919insTAG:p.A640delinsVA	Inframe	VUS(PM2_Supporting)	this study	mucopolysaccharidosis
		c.161A>G:p.N54S	Missense	VUS(PM2_Supporting)	this study	mucopolysaccharidosis
19,520	*PCCA*(NM_000282)	c.1284 + 1G>A	Splicing	P(PVS1+PS3+PM2_Supporting)	PMID: 24464666	Methylmalonic acidemia
		c.2002G>A:p.G668R	Missense	P(PS3+PM2_Supporting + PM3_Strong + PP3+PP4)	PMID: 27227689; 29978829	Methylmalonic acidemia
20,133	*PCCB*(NM_000532.5)	c.838dup:p.L280Pfs*11	Frameshift	P(PVS1+PM2_Supporting + PM3_Supporting + PP4)	PMID: 24863100	Methylmalonic acidemia
		c.184–2A>G	Splicing	P(PVS1+PM3+PM2_Supporting)	PMID: 15464417; 24863100	Methylmalonic acidemia
22,529	*MMUT* (NM_000255)	c.1106G>A:p.R369H	Missense	P(PS3+PM2+PM3)	PMID: 9929975; 17075691	Methylmalonic acidemia
		c.729_730insTT:p. D244Lfs*39	Frameshift	P(PVS1+PM2+PP3)	PMID: 23430940; 26454439	Methylmalonic acidemia
24,542	*MMACHC*(NM_015506)	c.609G>A:p.W203X	Nonsense	P(PVS1+PM2+PP3+PP4)	PMID: 19370762; 20631720	Cerebral palsy; pneumonia
		c.658_660del (p.Lys220del)	Inframe	P(PM2+PM3+PM4+PP3+PP4)	PMID: 20631720; 26563984	Cerebral palsy; pneumonia
24,329	*IVD* (NM_002225.5)	c.466-3_466-2delinsGG	Intronic variants	P(PVS1+PM2+PP3+PP4)	this study	Isovaleric acidemia
		c.1016G>A:p.C339Y	Missense	P(PM1+PM2+PP3+PP4)	this study	Isovaleric acidemia
25,492	*CFTR* (NM_000492)	c.650A>G:p.E217G	Missense	VUS(PM1)	PMID: 12952861; 11589722	Electrolyte disturbance
		c.374T>C:p.I125T	Missense	VUS(PM1)	PMID: 25869325; 16678503; 12439892	Electrolyte disturbance
26,431	*OTC*(NM_000531.6)	c.20delT (p.I7Tfs*3)	Frameshift	P(PVS1+PM2+PP4)	this study	Ornithine transcarbamylase deficiency
27,697	*MMUT* (NM_000255)	c.2179C>T:p.R727X	Nonsense	P(PM3_VeryStrong + PVS1_Strong + PM2+PP4)	PMID: 16490061; 16281286	Methylmalonic academia
		c.1280G>A:p.G427D	Missense	P(PM3_Strong + PM1+PM2+PP3+PP4)	PMID: 23430940; 25299208	Methylmalonic academia
29,112	*ASS1*(NM_054012)	c.847G>A:p.E283K	Missense	LP(PVS1+PM2_supporting)	PMID: 28111830; 23611581	Citrullinemia
		c.1048C>T:p.Q350X	Nonsense	LP(PM3_strong + PM2_supporting + PP1+PP3)	PMID: 18473344; 19006241	Citrullinemia
Nervous System						
19,738	*PRKDC*(NM_006904.7)	c.11507C>T (p.Pro3836Leu)	Missense	VUS	NA	Microcephaly
		c.10684T>A (p.Leu3562Met)	Missense	VUS	NA	Microcephaly
20,254	*SLC25A1*(NM_005984)	c.628C>T:p.R210X	Nonsense	P(PVS1+PM1+PM2+PM4)	PMID: 32660532	Neurodevelopmental deficits
		c.341A>T:p.D114V	Missense	LP(PM2+PM3+PP2+PP3)	this study	Neurodevelopmental deficits
20,519	*MTM1*(NM_000252)	c.1456C>T:p.R486X	Nonsense	P(PVS1+PM3+PM2_Supporting)	PMID: 12522554; 11,793,470; 10,063,835	Fetal intrauterine
		c.1456C>T:p.R486X	Nonsense	P(PVS1+PM3+PM2_Supporting)	PMID: 12522554; 11,793,470; 10,063,835	Fetal intrauterine
21,497	*PCLO*(NM_033026.5)	c.7790C>G:p.A2597G	Missense	VUS(PM2+PP4)	this study	Infantile Spasms
		c.10691G>A:p.S3564N	Missense	VUS(PP3+PP4)	NA	Infantile Spasms
24,542	*COQ4*(NM_016035)	c.402 + 1G>A	Splicing	LP(PVS1+PM2)	PMID: 25658047; 31,396,399	Cerebral palsy; pneumonia
		c.550T>C:p.W184R	Missense	LP(PM2+PM3+PP3+PP4)	PMID: 30109123; 31,396,399	Cerebral palsy; pneumonia
26,780	*PTPN23*(NM_015466)	c.4634C>T:p.P1545L	Missense	VUS(PM2)	this study	Neurodevelopmental deficits
		c.3901C>T:p.R1301C	Missense	VUS(PM2)	NA	Neurodevelopmental deficits
Cardiovascular System						
27,372	*TMEM67*(NM_153704)	c.539C>T:p.T180I	Missense	VUS(PM2)	NA	Cardiac dysplasia
		c.577–28C>T	Intronic variants	VUS(PM2)	this study	Cardiac dysplasia
28,156	*FANCA*(NM_000135)	c.3894G>A:p.R1298R	Samesense	VUS(PM2_Supporting)	this study	NA
		c.1799G>A:p.R600H	Missense	VUS(PM2_Supporting)	PMID: 31721781	NA
		c.1806G>A:p.V602V	Samesense	VUS(PM2_Supporting)	NA	NA
29,100	*CEP290*(NM_025114)	c.2144T>G:p.L715X	Nonsense	P(PVS1+PM2+PM3)	PMID: 27375279; 31,840,411	Cardiac dysplasia; Polycystic kidney disease
		c.2144T>G:p.L715X	Nonsense	P(PVS1+PM2+PM3)	PMID: 27375279; 31,840,411	Cardiac dysplasia; Polycystic kidney disease
Skeletal System						
20,298	*COL11A1*(NM_080630)	c.1245 + 1G>A	Splicing	P(PVS1+PM3+PM2_Supporting)	PMID:32756486; 32,427,345; 25,240,749	Skeletal dysplasia
	*PEX7*(NM_000288)	c.179delT:p.F61Lfs*13	Frameshift	P(PVS1+PS3+PM2_Supporting)	PMID:34671977; 34,229,749; 12,522,768	Skeletal dysplasia
		c.122G>C:p.G41A	Missense	LP(PM5+PM2+PP2+PP3)	this study	Skeletal dysplasia
26,936	*GFM1*(NM_024996.5)	c.80A>C:p.Q27P	Missense	VUS(PM2+PP3)	this study	Skeletal dysplasia
		c.1521G>T:p.R507S	Missense	VUS(PM1+PM2+PP3)	this study	Skeletal dysplasia
28,920	*ASCC1*(NM_001198798)	c.829C>T:p.H277Y	Missense	VUS(PM1+PM2_Supporting + PP3)	this study	Abnormal facial appearance; Skeletal dysplasia
		c.14G>C:p.R5P	Missense	VUS(PM2_Supporting + PP3)	this study	Abnormal facial appearance; Skeletal dysplasia
Kidney						
20,770	*PKHD1*(NM_138694)	c.10997T>G:p.I3666S	Missense	VUS(PM2_Supporting + PP3)	PMID: 31730820	abnormal renal function
		c.3860T>G:p.V1287G	Missense	VUS(PM2_Supporting)	PMID: 31813136; 31,730,820	abnormal renal function
		c.6408C>A:p.S2136R	Missense	VUS(PM2_Supporting + PP3)	PMID: 31730820	abnormal renal function
19,796	*DYNC2H1*(NM_001080463)	c.2170G>A:p.E724K	Missense	LP(PM3+PM2+PP1+PP4)	PMID: 22499340; 31,730,820	Polycystic kidney disease
		c.1288C>T:p.R430C	Missense	LP(PM2+PM3+PM5)	PMID: 22499340; 31,730,820	Polycystic kidney disease
27,169	*DHCR7*(NM_001360)	c.914A>G:p.Y305C	Missense	VUS(PM2+PP3)	this study	Polycystic kidney disease
		c.987C>T:p.P329P	Missense	VUS(PM2)	NA	Polycystic kidney disease
Skin						
20,337	*ALOX12B*(NM_001139)	c.2060A>G:p.Y687C	Missense	LP(PM1+PM2+PP3+PP5)	NA	Ichthyosis vulgaris
		c.1405C>T:p.R469W	Missense	LP(PM1+PM2+PM3)	PMID: 31046801	Ichthyosis vulgaris
24,371	*TYR* (NM_000372)	c.115T>C:p.W39R	Missense	VUS(PM2+PM3+PP3)	PMID: 19865097	Albinism
		c.832C>T:p.R278X	Nonsense	P(PVS1+PM2+PP3)	PMID: 22734612; 25,919,014	Albinism
Others						
25,240	*UBE3B*(NM_130466)	c.1447dup:p.T483Nfs*7	Frameshift	LP(PVS1+PM2+PP3)	this study	Polysomatous
		c.3065G>A:p.R1022H	Missense	VUS(PM1+PM2+PP3)	this study	Polysomatous
		c.2710C>T:p.R904C	Missense	VUS(PM1+PM2+PP3)	NA	Polysomatous
25,271	*ARL13B*(NM_001174150)	c.568A>G:p.I190V	Missense	VUS(PM1)	PMID: 27491411	Abdominal mass occupancy
		c.568A>G:p.I190V	Missense	VUS(PM1)	PMID: 27491411	Abdominal mass occupancy
27,022	*GJB2*(NM_004004)	c.109G>A:p.V37I	Missense	P(PS4+PM3+PP1_Strong)	PMID: 31160754; 17,935,238	Respiratory failure; dysphagia
		c.109G>A:p.V37I	Missense	P(PS4+PM3+PP1_Strong)	PMID: 31160754; 17,935,238	Respiratory failure; dysphagia
	*GAA* (NM_000152)	c.2132C>G:p.T711R	Missense	VUS(PM2+PP3+PM3_Supporting)	PMID: 28394184; 22,644,586; 30,275,481	Respiratory failure; dysphagia
		c.752C>T:p.S251L	Missense	LP(PM3+PM2+PS3_Supporting)	PMID: 18458862; 22,644,586	Respiratory failure; dysphagia
		c.761C>T:p.S254L	Missense	LP(PM3+PM2+PP3+PS3_Supporting)	PMID: 18458862; 22,644,586	Respiratory failure; dysphagia
	*SURF1*(NM_003172.4)	c.281dupT:p.L94Ffs*8	Frameshift	P(PVS1+PM2+PM3)	PMID: 24462369	Respiratory failure; dysphagia
		c.688C>T:p.R230X	Nonsense	P(PVS1+PM2+PM3)	PMID: 10558868; 25,525,159	Respiratory failure; dysphagia

Details of the reported variants in couples with an adverse pregnancy history in the present study. The description of the variants was based on the guidelines provided by the American College of Medical Genetics and Genomics (ACMG) and Association for Molecular Pathology (AMP).

^a^ Literature significantly associated with the variants or used in the variant interpretation is listed in the Justification. NA, means unavailable, and ‘this study’ indicates that we could not find the variant in ClinVar or the Human Gene Mutation Database (HGMD).

^b^ Previous clinical phenotypes of these aborted fetuses or prematurely deceased children are listed in abnormalities of the proband.

**TABLE 2:** Distribution of variants, pathogenic/likely pathogenic cases, and genes among clinical groups with adverse pregnancy history.

Clinical groups	A: variants		B: P/LP cases		C: genes	
	Number of variants	Frequency (%)	Number of P/LP variants	Frequency (%)	Number of genes	Frequency (%)
Metabolic system	23	31.94	19	42.24	11	32.35
Nervous System	12	16.67	6	14.29	6	17.65
Cardiovascular System	7	9.72	2	4.77	3	8.82
Skeletal system	7	9.72	3	7.14	4	11.76
Kidney	7	9.72	2	4.77	3	8.82
Skin	4	5.56	3	7.14	2	5.88
Others	12	16.67	7	16.67	5	14.71
Total	72	100	42	100	34	100

Note: Columns A, B, and C indicate the absolute number and frequency of all variants, P/LP, variants, and genes detected in the clinical subgroups, respectively. Note that the numbers in A and B are the actual number of detections, and the detections of the same mutation in different individuals are not combined. (Individual data are presented in Table 1.) A color scale was used to compare the distribution of frequencies, with a darker color indicating a greater distribution. Abbreviations: P/LP, pathogenic/likely pathogenic.

### 3.2 Variant detection characteristics

Of the 72 variants detected, the highest carrier rate for single variants was 4.17% for the known variant of *MMACHC*, NM_015506: exon4 c.609G>A, which is associated with methylmalonic aciduria and homocystinuria, cblC type (OMIM#277400), and the inheritance was autosomal recessive. The following variants were detected more than once: *MTM1*, NM_000252: exon13 c.1456C>T p.R486X (myopathy, centronuclear; OMIM#310400); *CEP290*, NM_025114: exon21 c.2144T>G p.L715 (Joubert syndrome 1; OMIM#213300), *ARL13B*, NM_001174150: exon5 c.568A>G p.I190V (Joubert syndrome 8; OMIM#612291), and *GJB2*, NM_004004: exon2, c.109G>A p.V37I (deafness, autosomal recessive 1A; OMIM220290). For a complete list of information on the variants, see Table 1.

### 3.3 Detection of variant genes and disease characteristics

The 72 detected variants belonged to 34 genes, with the highest proportion of these variants belonging to metabolic system-related genes at 32.4% (n = 11) and the second highest proportion belonging to genes involved in the nervous system (n = 6, 17.6%).; further details are presented in Table 2. Notably, both *MMACHC* and *MMUT*, which are associated with the metabolic system, were detected in both couples and had a variant rating of pathogenic. This finding suggests that *MMACHC* and *MMUT* should be considered priority genes in the metabolic system and ECS. After predictive analyses of variant gene-related diseases, the highest number of related diseases appeared to be methylmalonic aciduria and homocystinuria, cblC type (OMIM#277400; *MMACHC*), propionic acidemia (OMIM#606054; *PCCA* and *PCCB*), and methylmalonic aciduria, mut type (OMIM#251000; *MMUT*), which were detected in four cases.

### 3.4 General overview of WES in subjects with a family history of genetic diseases or consanguineous marriage

In our study, a total of 18 individuals with a family history of genetic diseases or consanguineous marriages were subjected to WES. After the WES data were analyzed, a total of 26 variants were detected. After ACMG rating, 11 variants were classified as P/LP (Table 3).In general, VUS were found in seven individuals and P/LP variants were found in 11, with a detection rate of 61.11%. In subjects with a family history of genetic diseases or a history of consanguineous marriage, the number of variants detected in each system and the number of P/LP variants detected did not differ much. Notably, *SYNE1* and *PKD1* were detected with variants in multiple families (Table 4) and two at-risk couples (ARCs; couples in which both partners carry P/LP variants in the same gene) with recessive pathogenic variants for *AMH* and *GJB2* (*AMH*: c.1165G>T and *GJB2*: c.-23 + 1G>A, respectively) were found.

**TABLE 3:** Distribution of variants, pathogenic/likely pathogenic cases, and genes among clinical groups with a family history of genetic diseases or consanguineous marriages.

Clinical groups	A-variants		B-P/LP cases		C-genes	
	Number of variants	Frequency (%)	Number of P/LP variants	Frequency (%)	Number of genes	Frequency (%)
Metabolic system	4	15.38	2	18.19	2	15.38
Nervous System	8	30.77	0	0	3	23.08
Skeletal system	5	19.23	2	18.19	5	38.46
Kidney	4	15.38	4	36.36	1	7.69
Auditory system	5	19.23	3	27.27	2	15.38
Total	26	100	11	100	13	100

Columns A, B, and C indicate the absolute number and frequency of all variants, P/LP, variants, and genes detected in the clinical subgroups, respectively. Note that the numbers in A and B are the actual number of detections, and the detections of the same mutation in different individuals are not combined. (Individual data are presented in Table 2.) A color scale was used to compare the distribution of frequencies, with a darker color indicating a greater distribution. Abbreviations: P/LP, pathogenic/likely pathogenic.

**TABLE 4:** Details of the variants identified in couples with a family history of genetic diseases or consanguineous marriages in the present study.

Case ID	Gene and Transcript	Variant	Type of variation	ACMG	Justification^a^	Abnormalities of the proband^b^
Audiological system						
27,448	*GJB2*(NM_004004.6)	c.-23 + 1G>A	Splicing	LP(PM3_Very Strong + PS3+PM2)	PMID: 21776002; 21,122,151; 16,380,907	consanguineous marriage
		c.-23 + 1G>A	Splicing	LP(PM3_Very Strong + PS3+PM2)	PMID: 21776002; 21,122,151; 16,380,907	consanguineous marriage
	*SYNE1*(NM_033071)	c.24746G>A:p.G8249E	Missense	VUS(PM2)	NA	consanguineous marriage
		c.23446G>A:p.V7816M	Missense	VUS(PM2)	NA	consanguineous marriage
		c.12138 + 2T>C	Splicing	P(PVS1+PM2)	NA	consanguineous marriage
Metabolic System						
26,742	*MLYCD*(NM_012213)	c.742G>A:p.E248K	Missense	VUS(PM1+PM2+PP3)	this study	consanguineous marriage
		c.742G>A:p.E248K	Missense	VUS(PM1+PM2+PP3)	this study	consanguineous marriage
	*AMH*(NM_000479)	c.1165G>T:p.E389X	Nonsense	P(PVS1+PM2)	PMID: 32172781; 31,277,073	consanguineous marriage
		c.1165G>T:p.E389X	Nonsense	P(PVS1+PM2)	PMID: 32172781; 31,277,073	consanguineous marriage
Nervous System						
23,157	*SYNE1*(NM_182961)	c.24586T>A:p.C8196S	Missense	VUS(PM2_Supporting + PP3)	NA	consanguineous marriage
	*F8*(NM_000132.4)	c.1097A>G:p.P366D	Missense	VUS(PM1+PM2_Supporting)	NA	consanguineous marriage
27,410	*ERCC6*(NM_000124.4)	c.2647C>T:p.L883F	Missense	VUS(PM2+PP3)	NA	Family history of Intellectual impairment
		c.2647C>T:p.L883F	Missense	VUS(PM2+PP3)	NA	Family history of Intellectual impairment
		c.2647C>T:p.L883F	Missense	VUS(PM2+PP3)	NA	Family history of Intellectual impairment
		c.2962A>G:p.K988E	Missense	VUS(PM2+PP3)	NA	Family history of Intellectual impairment
		c.2962A>G:p.K988E	Missense	VUS(PM2+PP3)	NA	Family history of Intellectual impairment
		c.2962A>G:p.K988E	Missense	VUS(PM2+PP3)	NA	Family history of Intellectual impairment
Skeletal System						
20,253	*CLCN7*(NM_001114331)	c.1141A>G:p.R381G	Missense	LP(PP3_Verystrong + PM2_Supporting)	NA	Family history of genetic skeletal disorders
	*FBN1*(NM_000138)	c.4152G>A:p.M1384I	Missense	LP(PM1+PM2+PP2+PP4)	NA	Family history of genetic skeletal disorders
22,129	*FAT1*(NM_005245)	c.3770G>A:p.R1257Q	Missense	VUS(PM2_Supporting)	PMID:25615407	consanguineous marriage
	*OBSL1*(NM_015311.3)	c.2135-3_2135-2delCA	Intronic variants	VUS(PM2_Supporting)	PMID:33107243	consanguineous marriage
	*AR* (NM_000044)	c.528C>A:p.S176R	Missense	VUS(PM1_Supporting)	PMID:32985417; 30,411,392; 28,624,954	consanguineous marriage
Kidney						
23,870	*PKD1*(NM_001009944)	c.10420C>T:p.Q3474X	Nonsense	P(PVS1+PM2_Supporting + PS3)	PMID:31740648; 31,730,820; 29,590,654	Family history of Polycystic kidney disease
25,060		c.4480_4481insCGTGGGC:p.R1494Pfs*31	Frameshift	LP(PVS1+PM2_Supporting)	NA	Family history of Polycystic kidney disease
25,939		c.12010C>T:p.Q4004X	Nonsense	P(PVS1+PM2_Supporting + PP1)	PMID:29590654; 30,413,633; 29,529,603	Family history of Polycystic kidney disease
28,641		c.6397_6399delTTC:p.F2133del	Inframe	P(PS4_supporting + PM2_supporting + PM4+PP1+PP4)	PMID:22367170	Family history of Polycystic kidney disease

Details of the reported variants of couples with a family history of genetic diseases or consanguineous marriages in the present study. All subjects with a history of consanguineous marriage were couples, and among subjects with a history of genetic disease, only 27,410 and 27,411 were a pair of couple. The description of the variants was based on guidelines provided by the American College of Medical Genetics and Genomics (ACMG) and Association for Molecular Pathology (AMP).

^a^ Literature significantly associated with the variants or used in the variant interpretation is listed in the Justification. NA, means unavailable, and ‘this study’ indicates that we could not find the variant in ClinVar or the Human Gene Mutation Database (HGMD).

^b^ Previous clinical phenotypes of these aborted fetuses or prematurely deceased children are listed in abnormalities of the proband.

### 3.5 Recessive disease prevalence estimation

**FIGURE 1:**
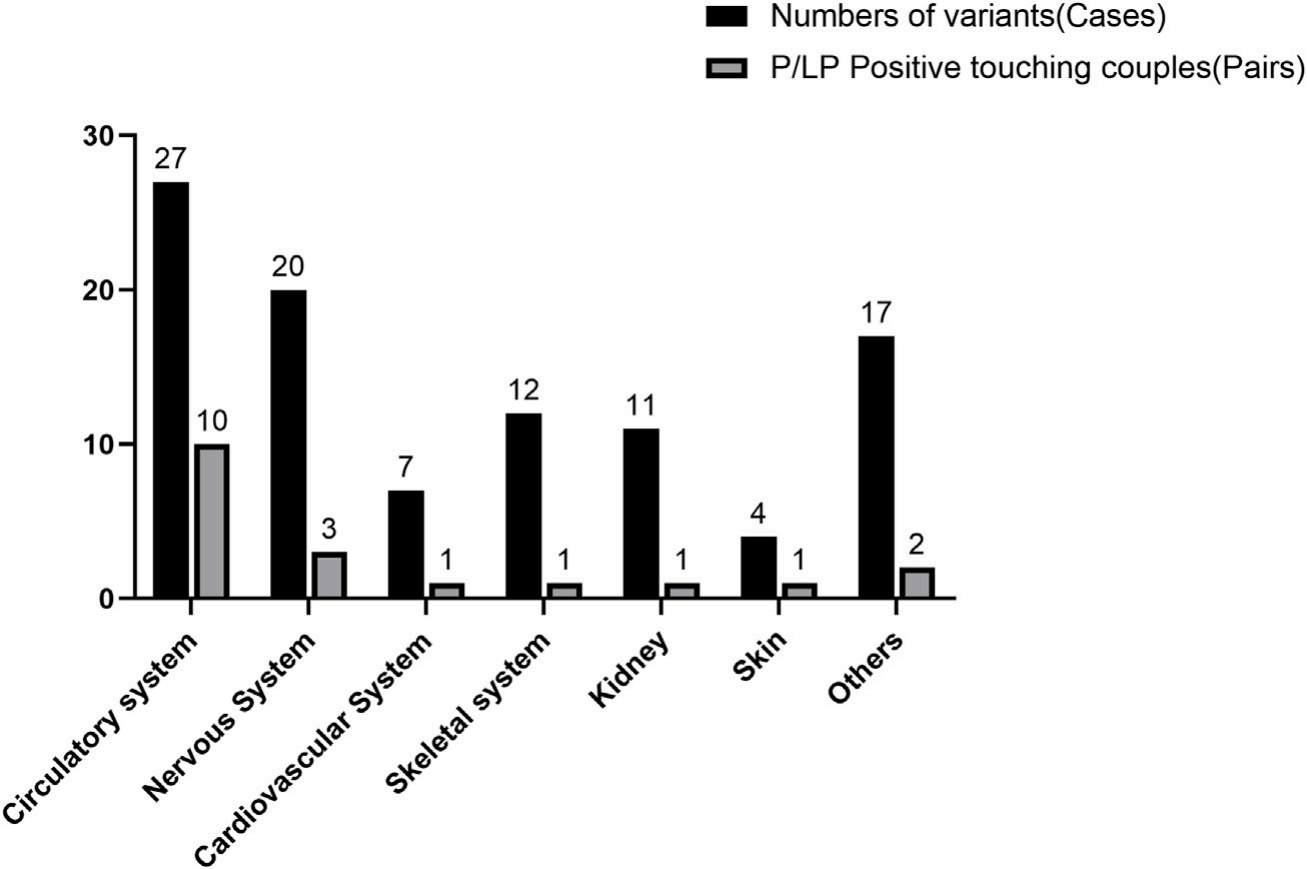
Frequency of variants in the entire cohort compared to the at-risk couples with pathogenic/likely pathogenic variants. The number of cases is shown directly above each bar for each category; the percentages above the number of cases or pairs of couples for each category are shown in parentheses.

For all subjects, P/LP variants were found in 47 individuals, and P/LP variants were found in 47, with an overall detection rate of 36.72%. The rate of reported variants per patient was calculated to be 0.633. In 81 cases (63.3%), we found candidate variants. However, according to the ACMG guidelines, only 47 cases (36.7%) could be classified as P/LP for the variants. When both individuals in a couple carry pathogenic variants in the same recessive gene (i.e., an ARC), the likelihood of giving birth to a child with a recessive disease is as high as 25%. Therefore, ARCs require a detailed prenatal or preimplantation diagnosis, which demands extra attention in clinical practice. After WES of 128 high-risk couples, 19 couples were found to be carriers of P/LP variants in the same recessive gene. Variants in metabolic system-related genes in the ARCs were the most common (52.6%, n = 10), followed by variants in nervous system-related genes in the ARCs (15.8%, n = 3); the variants of genes of other systems in the ARCs were detected almost equally or less frequently (Figure 1). Also, considering that an ARC with recessive pathogenic variants has a 25% chance to give birth to a child with the recessive disease, the theoretical value of offspring disease was 4.75 out of all 128 subjects included in this study, with a theoretical offspring prevalence rate of up to 7.42%.

## 4 Discussion

In clinical practice, couples with adverse pregnancy histories, such as miscarriages, usually begin with cytogenetic testing, such as karyotyping, of the products of the miscarriages or fetuses (probands). For example, in 2022, Williamson et al. tested 13 fetuses with ultrasound abnormalities for karyotypic abnormalities to guide prenatal diagnosis (Williamson et al., 1987). In recent years, clinical WES has increasingly been used to diagnose patients with suspected genetic disorders. In 2014, Yang et al. performed a molecular diagnosis using the WES of 504 patients, showing that approximately 30% of positive cases carried pathogenic variants (Yang et al., 2014). However, this process relies on having a proband sample.

In our study, we analyzed WES data from 128 high-risk couples with an adverse pregnancy history or a family history of genetic diseases or consanguineous marriages and examined the carriage of rare recessive single-gene disorders. Uniquely, these high-risk families lacked proband information or samples. At the same time, these families often have a need for a healthy fetus, so they are likely to be the prospective parents. In the study, we found that among couples with adverse pregnancy histories without a fetal sample, P/LP variants were found in 38 individuals, and VUS were found in 26, with a detection rate of 34.55%. Furthermore, among members with a family history of genetic diseases or consanguineous marriages, P/LP variants were found in 11 individuals, and VUS were found in 7, with a detection rate of 50.00%. Ultimately, P/LP variants were found in 47 individuals, and VUS were found in 34, with an overall detection rate of 36.72%. The rate of reported variants per patient was calculated to be 0.633, and nearly half (54.1%) of the reported variants were classified as P/LP using the ACMG rating system.

Similar to ECS, we screened carriers in high-risk couples. To the best of our knowledge, this is the first study to apply WES to CS in high-risk families without probands. The main difference between our study and other studies is the inclusion criteria. We selected families with a history of adverse pregnancies, genetic diseases, or consanguineous marriages without a proband sample. We performed WES on these couples to determine the presence of pathogenic variants. In 81 cases (63.3%), we found candidate variants. However, according to the ACMG guidelines, only 47 cases (36.7%) could be classified as P/LP for the variants, which was also the detection rate in our study. Compared to the results of the CS in southern China conducted by Chau et al., in 2022, 48.8% of the patients were carriers of one or more recessive genetic diseases (Quaio et al., 2021), our carrier rates differed significantly, which may be attributed to the disease type associated with the patient’s adverse pregnancy. We reported only the variants associated with the disease type of the previous adverse pregnancy rather than all of the pathogenic variants (which would still be reported if there were ARCs of other systems in the couple). At the same time, Quaio et al. selected patients with symptoms of Mendelian disease for their study, which somewhat increased the likelihood of detecting P/LP variants. Therefore, by sequencing the whole exome of high-risk couples and assigning ACMG ratings to the variants, we analyzed the sequencing results to speculate on the possible causes of previous adverse pregnancies and better guide high-risk couples to support ongoing reproductive planning.

CS of couples also yields information on carriers in specific areas or populations for better guidance in avoiding birth defects. In 2022, Tong et al. performed exome sequencing on 2234 couples and detected 94.9% of positive carriers of at least one disease pathogenic variant; the genes with the highest number of mutations detected were *GJB2* and *CFTR*, respectively. Following this screen, couples with severe diseases were ultimately more inclined to choose elective options, such as PGT, gamete donation, and adoption (Tong et al., 2022). In our study, the genes with the highest rate of P/LP variants were *MMACHC*, *MMUT*, *GJB2*, *SYNE1*, *AMH*, and *PKD1*, all detected four times. This finding suggests that preconception testing for these genes should be of high priority for high-risk families. Half of these six genes with the highest mutation frequencies were associated with the metabolic system (50%, n = 3, N = 6). At the same time, the metabolic system-associated variants were also the most numerous among all detected variants (27.6%, n = 27). Even in ARCs, the most detected mutations were in the metabolic system (52.6%, n = 10), which we hypothesized may be due to sampling bias: issues with the metabolic system in families with a history of adverse pregnancies tends to be detected early in life or the prenatal period and is targeted for sampling. This finding also highlights the metabolic system as the most crucial, requiring extra attention in the prenatal testing of high-risk couples.

After WES, we finally detected 19 couples with recessive pathogenic variants in ARCs and obtained a theoretical prevalence rate of up to 7.42%. This discovery is several times higher than both the 0.26% reported by Quaio et al., who screened 320 patients as carriers of recessive Mendelian diseases in 2021, and the 1% global prevalence of recessive monogenic disorders (Solomon et al., 2013; Quaio et al., 2021), suggesting that there is a considerable offspring recessive disease-causing gene morbidity in high-risk families. At the same time, we believe that this irregularity is also due to sampling bias: almost all our participants had a history of adverse pregnancy. The high predicted incidence of offspring in this study further reinforced the hypothesis that these couples, whom we defined as high-risk families, were at higher risk of having a child with birth defects and required medical intervention. We provided guidance for 19 couples with recessive pathogenic variants of ARCs on pregnancy and fertility, strongly recommending *in vitro* fertilization, prenatal testing, and preimplantation genetic diagnosis to improve reproductive outcomes. Couples with detectable dominant pathogenic variants were informed of the likelihood of disease risk and advised to undergo genetic counseling to reduce the incidence of disease in their offspring. Finally, we demonstrated that the carrier data derived from WES of couples from high-risk families without proband samples could provide effective and accurate guidance for the reproduction of such families and prevent birth defects. Overall, our study effectively utilized WES data to guide future fertility practices in high-risk couples without a proband by collecting peripheral blood from both high-risk partners, performing WES, and grading candidate variants using the ACMG system. Even without a proband for validation, we demonstrated that CS of high-risk family members using WES could broadly and unambiguously identify causative variants for further precise genetic counseling.

Our study confirms that through the high detection of relevant pathogenic variants in parents and the very high proportion of ARCs, our screening strategy can be used for high-risk couples who lack a proband sample. Prospective parents would benefit from their carrier status through WES sequencing of parents in high-risk families, to determine their reproductive risks, and to make informed decisions. Our screening strategy may not only elucidate possible etiologic factors for deceased probands in these families but may also provide future fertility guidance and advice for high-risk parents. However, our study had some limitations, such as the inability to accurately diagnose the cause of prior adverse pregnancies. Additionally, our screening process for variants may be lengthy and inaccurate in cases where there is a lack of clinical information about the proband. Finally, WES technology also has some limitations, including a significant reduction in the ability to analyze the presence of pseudogenes, copy number variations, and large segments of homologous sequences (Saunders et al., 2012; Jelin and Vora, 2018). Single-molecule real-time (SMRT) sequencing has already been used for specific diseases to overcome the complex problems associated with NGS (Liang et al., 2022; Liu et al., 2022; Liang et al., 2023; Liu et al., 2023). As such, in the future, our results will be more instructive if we can supplement WES data with SMRT sequencing data for specific genes of high-risk family members, based on the clinical information of prevalent patients (Conlin et al., 2022).

## Data Availability

The datasets presented in this study can be found in online repositories. The names of the repository/repositories and accession number(s) can be found below: https://ngdc.cncb.ac.cn/gsa-human/, HRA007137.
