# Supplementary material for: Application of whole exome sequencing in carrier screening for high-risk families without probands
Source: Front Genet. 2024 Jun 24;15:1415811. doi: 10.3389/fgene.2024.1415811 (PMC11228263; doi:10.3389/fgene.2024.1415811)
Supplement: Supplementary file 1 [file Table1.DOCX]

**Table S1**

Adverse pregnancy history family obstetrics history

| Cases ID | premature or abortions | Born child | Pregnancy over second trimester | Anomalies in prior premature or abortions |
| --- | --- | --- | --- | --- |
| 17184 | 1 | — | — | Methylmalonic acidemia or propionic academia |
| 17587 | NA | NA | NA | Phenylketonurics |
| 17818 | 1 | — | — | Polycystic kidney disease |
| 18876 | NA | NA | NA | mucopolysaccharidosis |
| 20770 | 2 | 1 | 2 | abnormal renal function |
| 19738 | 3 | — | 3 | Microcephaly |
| 19796 | 2 | — | — | Polycystic kidney disease |
| 20254 | 2 | — | 2 | Neurodevelopmental deficits |
| 20337 | — | 1 | 1 | Ichthyosis vulgaris |
| 19520 | 2 | — | 2 | Methylmalonic acidemia |
| 20133 | 2 | — | 2 | Methylmalonic acidemia |
| 20298 | 1 | 1 | 2 | Skeletal dysplasia |
| 20519 | 2 | — | 2 | Fetal intrauterine |
| 21497 | 1 | — | 1 | Infantile Spasms |
| 22529 | 2 | — | 2 | Methylmalonic acidemia |
| 24371 | 1 | — | 1 | Albinism |
| 25240 | 2 | — | — | Polysomatous |
| 25271 | 1 | 1 | 2 | Abdominal mass occupancy |
| 24542 | 2 | — | 2 | Cerebral palsy; pneumonia |
| 24992 | 2 | — | 2 | Methylmalonic acidemia |
| 24329 | 1 | — | 1 | Isovaleric acidemia |
| 25492 | 1 | 1 | 2 | Electrolyte disturbance |
| 26431 | 1 | 1 | 2 | Ornithine transcarbamylase deficiency |
| 26780 | 2 | — | 2 | Neurodevelopmental deficits |
| 26936 | 3 | — | — | Skeletal dysplasia |
| 27022 | 3 | — | — | Respiratory failure; dysphagia |
| 27169 | 2 | — | — | Polycystic kidney disease |
| 27372 | 3 | — | — | Cardiac dysplasia |
| 27697 | 1 | — | — | Methylmalonic academia |
| 28156 | 3 | — | — | — |
| 28920 | 2 | — | — | Abnormal facial appearance; Skeletal dysplasia |
| 29100 | 2 | — | — | Cardiac dysplasia; Polycystic kidney disease |
| 29112 | 1 | — | — | Citrullinemia |

Only families with relevant variants detected are shown in the table and the ID number is the mother's number. NA means we do not know the exact number of previous abnormal pregnancies, only that there has been a history of adverse pregnancies with a particular systemic disease.

**Table S2**

Family history of hereditary disease

| Case ID | Family history of disease | relevant variants detected? |
| --- | --- | --- |
| 20253 | genetic skeletal disorders | Yes |
| 23870 | polycystic kidney disease | Yes |
| 25060 | polycystic kidney disease | Yes |
| 25939 | polycystic kidney disease | Yes |
| 27410 | Intellectual disability | Yes |
| 27411 | Intellectual disability | Yes |
| 27412 | Intellectual disability | Yes |
| 27413 | Intellectual disability | Yes |
| 28641 | polycystic kidney disease | Yes |
| 28960 | Schizophrenia | No |
